# PS-15 - THE PRINCIPAL HEALTH PROTECTION PRACTITIONER (PHPP) NETWORK: STRATEGIC SYSTEM LEADERSHIP STRENGTHENING HEALTH PROTECTION PRACTICE AND DRIVING QUALITY IMPROVEMENT ACROSS UKHSA HEALTH PROTECTION IN REGIONS (HPIR)

**DOI:** 10.1093/pubmed/fdag046.062

**Published:** 2026-07-09

**Authors:** Ms Laura Pomeroy, Ms Cassie Gregory, Ms Anita Turley, Ms Suzanne Calvert, Ms Rachael Kain, Ms Penelope Edwards, Mr Mohammud Edoo, Ms Joanna Keal, Ms Sarah Dowle, Ms Elizabeth Knapper, Ms Kate McPhedran, Ms Jaime Morgan, Mr Robert Seremani, Ms Valerie Unsworth, Ms Luisa Walker

**Affiliations:** UK Health Security Agency; UK Health Security Agency; UK Health Security Agency; UK Health Security Agency; UK Health Security Agency; UK Health Security Agency; UK Health Security Agency; UK Health Security Agency; UK Health Security Agency; UK Health Security Agency; UK Health Security Agency; UK Health Security Agency; UK Health Security Agency; UK Health Security Agency; UK Health Security Agency

## Abstract

**Background:**

The Principal Health Protection Practitioner (PHPP) role varies across Regions in line with local business models, but retains consistent core functions. PHPPs are members of Regional Senior Leadership/Management Teams and provide professional leadership to Health Protection Practitioners (HPPs), the largest workforce group in Health Protection Teams. They operate at a senior strategic and operational level, with portfolios that may include acute response leadership, stakeholder engagement, quality and governance, workforce and HR, learning and development, and specialist areas such as infection control, safeguarding, tuberculosis and care homes. PHPPs provide system leadership for health protection across England, champion professional standards, and support workforce development, working collaboratively to deliver national and regional outcomes.

**PHPP Network:**

Established in 2021, the PHPP Network enables pan-regional, cross-boundary collaboration and strategic system leadership. It consists of 14 PHPPs representing 9 Regions, with a rotating Chair (612 months) and diverse professional backgrounds including nursing and environmental health. The Network meets twice monthly virtually and twice yearly in person. Its scope includes peer support, shared learning, cross-regional project collaboration, and providing a forum to escalate issues and recommendations nationally. It also offers professional leadership and advocacy for the HPP workforce, promotes consistency and best practice while recognising regional variation, and drives quality improvement and change. An annual workplan guides activity, including commissioned work from Regional Senior Leadership Teams.

**PHPP Value and Impact:**

The PHPP Network strengthens professional leadership, enhances collaboration, and ensures a consistent, high-quality approach to health protection across England. It acts as a key reference point for the HPP profession, supporting workforce development and influencing national priorities. The Network has delivered tangible outputs, including review of HPP job descriptions to support recruitment and business needs, and mapping and review of out-of-hours functions. Overall, it enables coordinated system leadership, supports continuous improvement, and drives effective, responsive health protection services.

